# Association of Transmucosal Emergence Geometry and Peri‐Implant Diseases Prevalence Around Bone‐ and Tissue‐Level Implants: A Cross‐Sectional Study

**DOI:** 10.1111/clr.70131

**Published:** 2026-04-12

**Authors:** Clemens Raabe, Manrique Fonseca, Vivianne Chappuis, Gustavo Avila‐Ortiz, Diogo Moreira Rodrigues, Pablo Galindo‐Moreno, Emilio Couso‐Queiruga

**Affiliations:** ^1^ Department of Oral Surgery and Stomatology, School of Dental Medicine University of Bern Bern Switzerland; ^2^ Department of Oral Surgery and Implantology Goethe University Carolinum Frankfurt am Main Germany; ^3^ Department of Reconstructive Dentistry and Gerodontology, School of Dental Medicine University of Bern Bern Switzerland; ^4^ Department of Periodontics and Oral Medicine University of Michigan School of Dentistry Ann Arbor Michigan USA; ^5^ Department of Periodontology National Institute of Dental Sciences (INCO 25) Niterói Rio de Janeiro Brazil; ^6^ Department of Oral Surgery and Implant Dentistry, School of Dentistry University of Granada Granada Spain; ^7^ Instituto Biosanitario (IBS) Granada Granada Spain

**Keywords:** alveolar bone loss, clinical trial, dental implants, digital imaging, guided bone regeneration, image processing, osseointegration

## Abstract

**Objectives:**

This cross‐sectional study primarily aimed at investigating the association between the transmucosal emergence geometry and the prevalence of peri‐implant diseases in bone‐ and tissue‐level implants (BL/TL). As secondary objectives, the association of other implant‐, prosthesis‐, and patient‐related variables with crestal bone levels (CBL) and additional radiographic findings was explored.

**Material and Methods:**

Patients with non‐molar single tooth implant‐supported prostheses were retrospectively identified for inclusion. Clinical and radiographic examinations were performed cross‐sectionally to assess peri‐implant diseases (peri‐implant mucositis, peri‐implantitis), emergence geometry at the transmucosal region (profile type, emergence angles at two levels, and prosthetic platform height), and peri‐implant radiographic variables, including CBL. Data were analyzed using multivariate regression.

**Results:**

A total of 332 implants (166 BL/166 TL) in 266 patients (60 ± 17 years) were evaluated at a mean of 11.2 ± 1.5 years after implant placement. Peri‐implant mucositis was significantly influenced by the crown emergence angle (Level 2; OR = 1.03, *p* = 0.01) and peri‐implantitis by implant diameter (OR = 3.82, *p* < 0.0001). Crown emergence angles > 30° in BL implants and > 48° in TL implants were consistently associated with peri‐implant diseases. Mean CBL was 0.54 ± 1.34 mm, with lower values in TL (−0.24 mm; *p* = 0.036), females (−0.27 mm, *p* = 0.029), and implants with greater platform height (−0.175 mm/mm, *p* = 0.01). Peri‐implant sheathing was detected in 13.4% of implants, showing no association with implant‐related characteristics.

**Conclusions:**

Wider crown emergence angles were associated with peri‐implant diseases, whereas wider implant diameters were associated with increased peri‐implantitis prevalence. TL implant design and greater platform height were associated with reduced CBL.

## 1 Introduction

Implant therapy has evolved into a highly predictable and broadly adopted option for tooth replacement, with long‐term success hinging on sustained osseointegration and the health and stability of peri‐implant hard and soft tissues (Buser et al. 2017; Laleman and Lambert 2023; Schwarz et al. 2018).

However, crestal bone loss, defined as the vertical reduction of peri‐implant bone support, is frequently observed following implant placement (Albrektsson et al. 1986). Crestal bone loss can be physiologic, representing the expected bone remodeling that occurs after insertion of the final implant‐supported prosthesis (ISP), or pathologic, when the amount of bone loss exceeds the anticipated range of physiologic remodeling (Moser et al. 2026). While crestal bone loss does not always progress after its onset, early bone loss within 6 months after loading beyond approximately 0.5 mm has been strongly associated with continued progression, which may ultimately lead to implant failure in some cases (Galindo‐Moreno et al. 2022). Progressive bone loss is thought to result primarily from the presence of dysbiotic microbial biofilm and its byproducts, which elicit a destructive immunoinflammatory response in susceptible hosts, but it may be initiated and further modulated by other implant‐ and prosthetic‐related, site‐specific, systemic, and behavioral factors (Cafferata et al. 2025; Kumar et al. 2018). Among these factors, the geometry of the transmucosal emergence of the implant complex may influence functional load distribution and microbial biofilm retention and maturation, potentially predisposing for the onset and progression of peri‐implant diseases (Schwarz and Ramanauskaite 2022).

Dental implant design has evolved substantially over the years. Nowadays, implant fixtures are generally categorized according to their macroscopic design as either bone‐level (BL) or tissue‐level (TL) implants, based on the position of the implant‐abutment interface with respect to the crestal bone at the time of placement. Normally, in BL implants, the platform is at or apical to the alveolar bone crest, which provides clinicians greater flexibility to contour the features of the transmucosal component of the ISP according to the features of the peri‐implant tissues and the treatment needs (Del Amo et al. 2024; Spinato et al. 2018). In contrast, in TL implants, the platform is normally placed coronal to the alveolar bone crest, which implies that part of the transmucosal component is incorporated within the fixture itself, simplifying restorative workflows but limiting individual customization (Katafuchi et al. 2018; Mattheos et al. 2021). The implant‐abutment interface, where the prosthesis is connected to the fixture, may serve as a niche for microbial biofilm. Therefore, the role of the marginal discrepancy between the abutment and the implant fixture, the so‐called microgap, and its proximity to the bone crest in the onset and progression of crestal bone loss and peri‐implant diseases has been widely debated (Liu and Wang 2017).

The geometric features of the transmucosal emergence have also been recognized to potentially influence the onset and progression of crestal bone loss and the occurrence of peri‐implant diseases. A recent meta‐analysis reported that ISPs with emergence angles exceeding 30° and convex profiles were associated with a higher risk of peri‐implantitis, particularly in BL implants (Lin et al. 2025). However, another recent randomized‐controlled trial and a meta‐analysis found no significant association between emergence angles and crestal bone loss (Atieh et al. 2023; Barwacz et al. 2025).

Implant diameter represents another fundamental parameter that can largely influence treatment planning and long‐term outcomes of implant therapy. Driven by the pursuit of minimally invasive protocols, narrow‐diameter implants (NDIs; ≤ 3.5 mm) have gained popularity to minimize the need for ridge augmentation, decrease surgical morbidity, and shorten overall treatment time (Raabe et al. 2024; Schiegnitz and Al‐Nawas 2018). Nevertheless, the reduced dimensions of NDIs limit the available surface area for osseointegration and may increase susceptibility to micro‐deformation or fracture under functional load, concentrating mechanical stresses at the bone‐to‐implant interface, potentially compromising osseointegration (Qiu et al. 2024). Although both BL and TL configurations, across narrow and standard diameters, exhibit high survival rates, their long‐term comparative effects on crestal bone levels (CBL) and peri‐implant disease prevalence remain insufficiently characterized (Lago et al. 2023; Vouros et al. 2012).

Accordingly, the primary aim of this cross‐sectional study was to investigate whether the transmucosal emergence geometry is associated with peri‐implant disease prevalence in single BL and TL implants placed in non‐molar sites. As a secondary objective, the association of other implant, prosthesis, and patient‐related variables with CBL and additional radiographic findings was explored.

## 2 Material and Methods

### 2.1 Study Design, Ethical Approval, and Setting

This study was designed as a single‐center, cross‐sectional investigation and is a sub‐analysis of a cohort described elsewhere (Couso‐Queiruga, Fonseca, et al. 2025). Ethical approval was obtained from the local institutional review board (KEK‐BE: 2023‐01651, Cantonal Ethics Commission [Kantonale Ethikkommission], Bern, Switzerland). The study adhered to the principles outlined in the Declaration of Helsinki (2024) and complied with the STROBE (Strengthening the Reporting of Observational Studies in Epidemiology) guidelines (Cuschieri 2019; World Medical Association 2025). Data collection was carried out in the Department of Oral Surgery and Stomatology, School of Dental Medicine, University of Bern, Switzerland, between November 2023 and May 2025.

### 2.2 Eligibility Criteria

The health records of all patients who underwent implant placement between January 2011 and November 2014 at the Department of Oral Surgery and Stomatology, School of Dental Medicine, University of Bern, Switzerland, were retrospectively screened for potential inclusion. Eligible patients were partially edentulous individuals aged ≥ 18 years, with either BL or TL implants (Straumann AG, Basel, Switzerland) featuring a hydrophilic micro‐rough surface (SLActive) and diameters of either 3.3 mm or 4.1 mm in function. Implants were placed in non‐molar single‐tooth sites and restored with either cement‐ or screw‐retained ISPs. Patients were invited to attend a clinical and radiographic examination via telephone or written correspondence. Written informed consent was obtained from all participants after a detailed explanation of the study objectives and procedures, with opportunities provided to address any questions.

### 2.3 Surgical and Restorative Procedures

All implant surgeries and related procedures (i.e., bone augmentation) were performed under local anesthesia by supervised residents and/or faculty members in the Department of Oral Surgery and Stomatology, School of Dental Medicine, University of Bern, Switzerland. For BL implants, the platform, and for TL implants, the junction between the machined and micro‐rough surfaces was placed at least 0.5 mm below the crestal bone level (Buser and Von Arx 2000). The final ISPs were inserted by the referring restorative dentists after a minimum healing period of 8 weeks.

### 2.4 Variables of Interest

**FIGURE 1:**
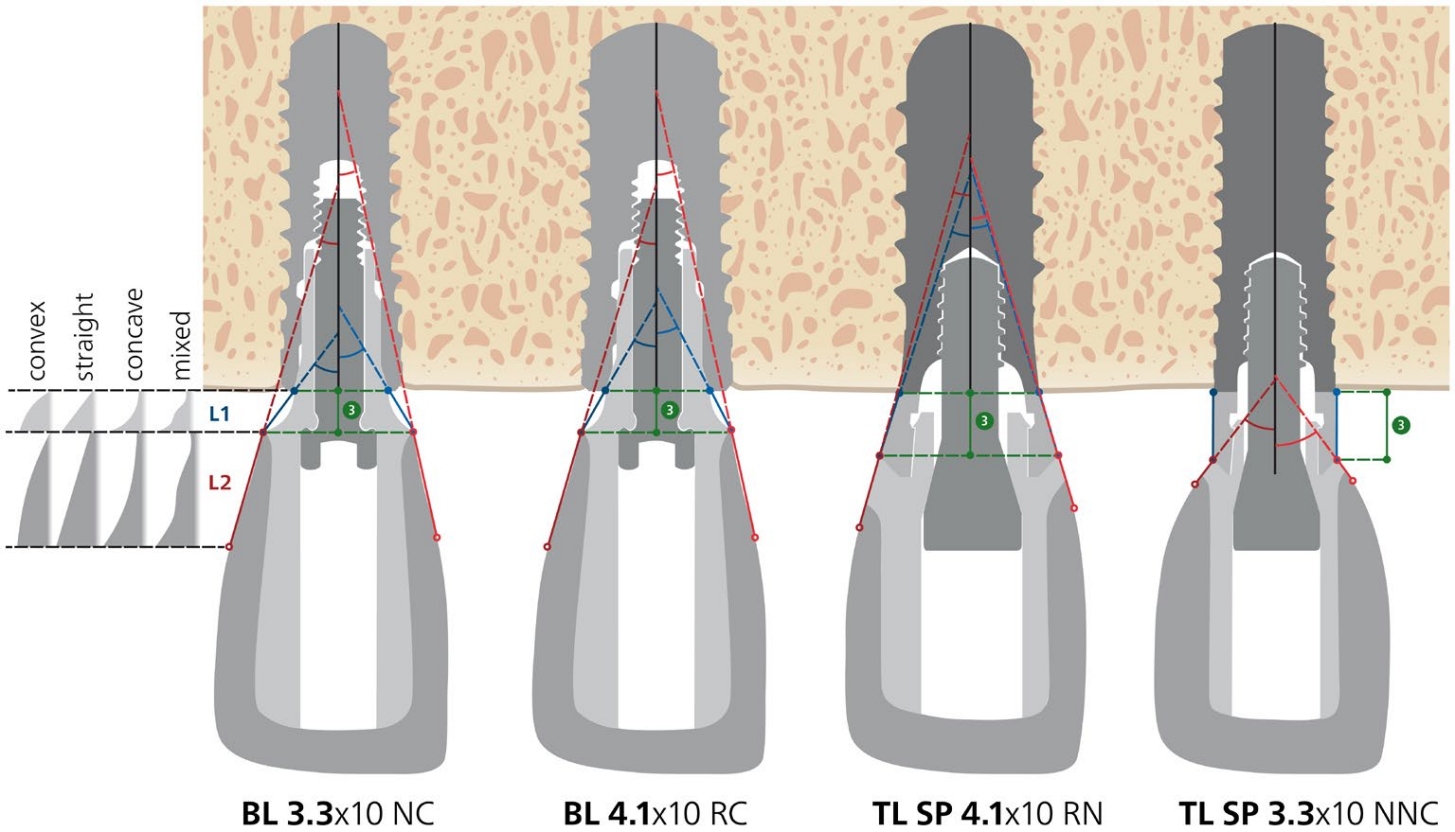
Bone‐level (BL) and tissue‐level (TL) implant designs in two diameters, illustrating the two assessment levels (L1, L2) for emergence profile and angle in relation to the implant's long axis. The abutment/collar emergence angle (L1, blue) is referenced to the implant platform in BL implants or to the transition between the machined and micro‐rough surface in TL implants. The crown emergence angle (L2, red) is referenced to the abutment platform in BL implants or to the implant platform in TL implants. Additionally, the prosthetic platform height (3, green) was measured, corresponding to the abutment height (BL) or the machined collar height (TL).

**FIGURE 2:**
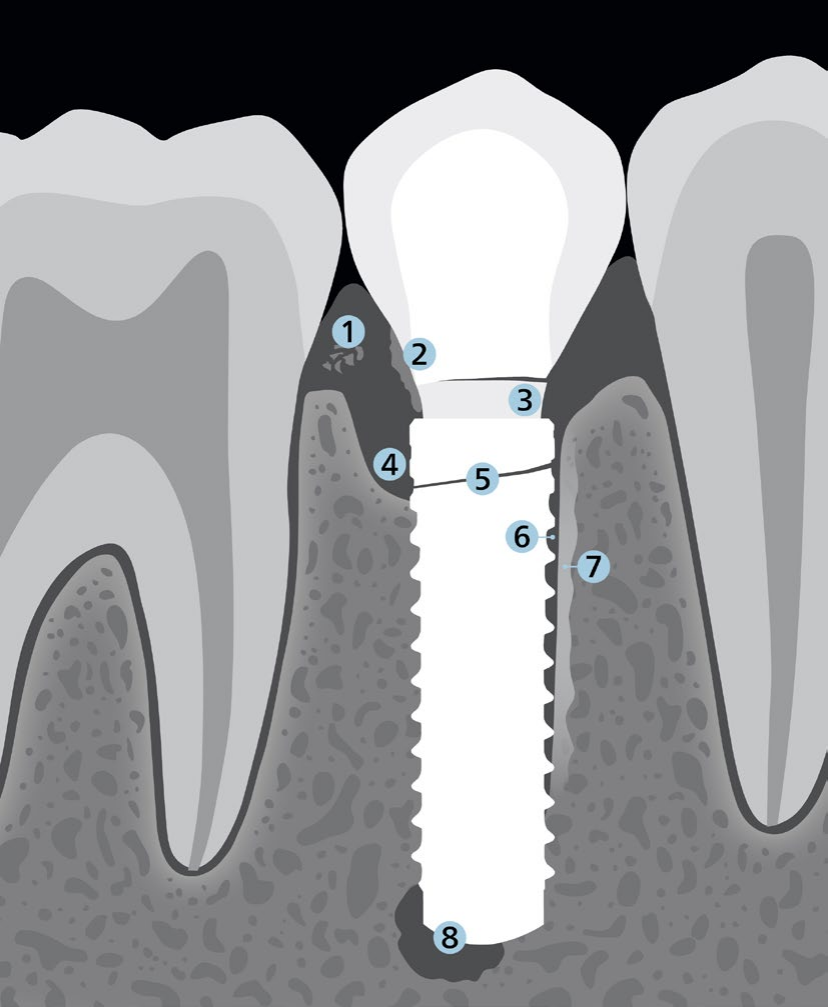
Radiographic findings of interest: (1) residual bone graft substitute particles in the peri‐implant soft tissues, (2) residual cement, (3) misfit of the implant‐supported prosthesis, (4) crestal bone loss, (5) implant fracture, (6) peri‐implant sheathing, (7) peri‐implant corticalization, and (8) retrograde peri‐implantitis.

- Diagnosis of peri‐implant mucositis and peri‐implantitis was based on the 2017 World Workshop Classification in Periodontal and Peri‐Implant Diseases and Conditions (Berglundh et al. 2018) according to the EFP S3 level clinical practice guideline on prevention and treatment of peri‐implant diseases (Herrera et al. 2023) as follows:
  ○ Peri‐implant health: absence of clinical signs of inflammation, a maximum of one single spot with bleeding on probing (BOP), no suppuration on probing (SOP), and absence of bone loss beyond crestal bone level changes resulting from initial bone remodeling after delivery of the definitive ISP.
  ○ Peri‐implant mucositis: presence of more than one spot with BOP or presence of a line of bleeding or profuse bleeding at any location and/or SOP, in the absence of bone loss beyond crestal bone level changes resulting from initial bone remodeling.
  ○ Peri‐implantitis: presence of BOP and/or SOP, PDs ≥ 6 mm, and bone levels ≥ 3 mm apical to the most coronal portion of the intraosseous part of the implant.
- Transmucosal emergence geometry, including profile type (i.e., concave, convex, straight, or mixed), emergence angles, and platform height, as shown in Figure 1
- Implant design (BL/TL) and diameter (3.3 mm/4.1 mm)
- Peri‐implant CBL and presence of other radiographic findings, as displayed in Figure 2.

### 2.5 Clinical and Radiographic Data Acquisition

A calibration session was conducted before the clinical evaluations to review the study protocol and establish uniform criteria for clinical assessment. To ensure reproducibility, the two experienced examiners (C.R. and E.C‐Q.) jointly evaluated five randomly selected participants. The comprehensive clinical evaluation was performed using a periodontal probe (Marquis probe; Hu‐Friedy, Chicago, IL) for diagnostic purposes, as previously described in a study involving part of this cohort (Braun et al. 2025).

Digital periapical radiographs were acquired (Xios XG Supreme Size 2, Dentsply Sirona, Charlotte, USA). The radiographs were centered on the implant using the parallel technique with standardized stock film holders (XCP, Dentsply Sirona). Radiographic data were independently analyzed by two examiners (C.R. and E.C‐Q.) using an open‐source software (ImageJ version 2, U.S. NIH, Bethesda, MD, USA), following an inter‐examiner calibration on radiographs obtained from the first 10 consecutive patients. For the digital evaluation, the software was calibrated using the thread distance of the respective implant design. CBL was assessed on the mesial and distal aspects in millimeters as the distance from the implant platform to the first bone‐to‐implant contact (Buser et al. 1990). For TL implants, the transmucosal machined collar height was subtracted from this measurement. Additionally, as shown in Figure 1, the angle and the profile of the transmucosal component were evaluated at two levels: (L1) abutment/collar emergence angle, at the level of the implant relative to the crestal bone (BL: implant platform, TL: border between machined/microrough surface), and (L2) crown emergence angle, at the level of the prosthetic platform (BL: abutment‐crown interface, TL: implant platform), based on a previously published methodology (Katafuchi et al. 2018). Crown and implant length were measured to the implant platform (anatomical) or the crestal bone level (clinical) to calculate the corresponding crown‐to‐implant ratios (CIR) (Laney et al. 2007; Tawil et al. 2006). Additionally, the prosthetic platform height was measured and recorded. For BL implants, this corresponded to the height of the transmucosal abutment between the implant platform and the crown margin. In the TL group, this was the machined collar height. Finally, aside from CBL, other parameters such as the presence of residual bone graft substitute particles, cement remnants, the fit of the ISP, peri‐implant sheathing or corticalization, implant fracture, and retrograde peri‐implantitis were also assessed (Sahrmann et al. 2024), as shown in Figure 2.

Additional details on data acquisition in this patient cohort have been previously published (Couso‐Queiruga, Fonseca, et al. 2025).

### 2.6 Statistical Analysis

All analyses were performed with a specific software package (R version 4.3.2), using packages “tidyverse”, “lme4”, “lmerTest”, “robustlmm”, “geepack”, “DHARMa” and “DescTools” (R Core Team 2020). General characteristics were summarized as means, medians, and standard deviations for continuous variables and as frequencies and percentages for categorical variables. Profile type, emergence angles at L1 and L2, and radiographic findings, including CBL, were summarized in the same manner, stratified by site (mesial/distal), implant design, and diameter.

Intraclass correlation coefficients (ICCs) were calculated to assess inter‐rater agreement for CBL and angles at L1 and L2, and were further classified by Cicchetti's grading scale (Cicchetti 1994). The means between both examiners were used for further analyses.

As the primary models showed violation of normality of residuals and the presence of outliers, robust estimation by Koller was applied (Koller 2016). When assessing dichotomous outcomes (adverse events: “yes”/“no”) and peri‐implant diseases (“healthy” vs. “peri‐implantitis”, “healthy” vs. “peri‐implant mucositis”), mixed logistic regression models or, in case of an insufficient model fit, generalized estimating equations (GEE) logistic regression models were used where repeated measurements were modeled as “exchangeable”.

In order not to overparameterize models (peri‐implant diseases) or to distinguish between (highly) correlated parameters (peri‐implant diseases and CBL), parameters of interest, implant design, diameter, profile type and emergence angle were first screened in an univariate analysis. In case of significance, they were assessed in a multivariate context by adding other biologically plausible correcting factors like gender, crown‐to‐implant ratio, platform height, etc. As profile levels and emergence angles strongly correlated, their effects were calculated in separate models. The assessment of emergence angles was done directly in a multivariate context.

Goodness‐of‐fit of final linear models was further assessed with the methods by Hartig and by checking for collinearity (multivariate models only; Hartig 2024). Goodness‐of‐fit of logistic models was again assessed with the help of the DHARMa package and by using the le Cessie‐van Houwelingen‐Copas‐Hosmer test (Hosmer et al. 1997).

All *p*‐values equal to or less than 0.05 were considered statistically significant.

### 2.7 Sample Size Calculation

To determine the required sample size for a two‐group comparison of a dichotomous endpoint, data from a previous study were used (Katafuchi et al. 2018). In that study, the prevalence of peri‐implantitis in BL and TL implants with a mesial and/or distal emergence angle > 30 degrees was 31.3% and 7.7%, respectively. Based on these estimates, a sample size calculation was conducted for a two‐sided test with 95% statistical power and a significance level of 0.05, indicating that a minimum of 70 implants per group would be required to reliably detect a difference in event rates. Considering that the effect of implant diameter was also a variable of interest, ≥ 70 implants in each combination of implant design and diameter (2 × 2 design) would be necessary; 166 implants were included in each group (BL vs. TL).

## 3 Results

### 3.1 Sample Characteristics

A total of 332 implants in 266 patients (132 females/134 males, mean age: 60 ± 17 years) were evaluated after a mean of 11.2 ± 1.5 years following implant placement. The sample included 166 BL and 166 TL implants, evenly distributed between diameters of 3.3 mm and 4.1 mm. Implant lengths were 8 mm (*n* = 25), 10 mm (*n* = 172), 12 mm (*n* = 120), and 14 mm (*n* = 15). Implants were placed in the central incisor (*n* = 76), lateral incisor (*n* = 74), canine (*n* = 18), first premolar (*n* = 75), and second premolar (*n* = 89) regions, in either the maxilla (*n* = 255) or the mandible (*n* = 77). The surgical procedures included standard implant placement (*n* = 70), as well as implant placement with simultaneous (*n* = 233), or staged horizontal bone augmentation (*n* = 8), or sinus floor elevation (*n* = 30). ISPs were either cemented (*n* = 48) or screw‐retained (*n* = 284). Details on the subgroup are displayed in Table S1.

The mean emergence angles at L1 and L2 were 26° ± 7° and 16° ± 8° for BL and 7° ± 7° and 25° ± 12° for TL. Regarding profile type, it was concave, convex, straight, or mixed in 63.3%, 3.6%, 33.1%, and 0% in BL versus 50%, 0%, 50%, and 0% in TL at L1. At L2, the respective distributions were 25%, 38.1%, 35.7%, and 1.2% in BL versus 22.6%, 42.2%, 34.3%, and 0.9% in TL. The mean prosthetic platform height was 1.52 ± 0.91 mm in BL versus 1.94 ± 0.35 mm in TL implants.

At the implant level, the prevalence of peri‐implant health, peri‐implant mucositis, and peri‐implantitis was 20.5%, 70.5%, and 9.0% for TL implants, and 21.1%, 67.5%, and 11.4% for BL implants, respectively. For NDI, the corresponding rates were 24.1%, 68.7%, and 7.2%, while for SDI, they were 17.5%, 69.2%, and 13.3%. Mean CBL was 0.54 ± 1.34 mm. Detailed subgroup data are displayed in Table 1. The ICCs for CBL and angle measurements ranged from 0.89 to 0.98 (95% CI: 0.87 to 0.98), indicating high inter‐rater reliability (Table *2*).

**TABLE 1:** Summary of crestal bone level, transmucosal emergence geometry, and peri‐implant tissue diagnosis by implant design and diameters. Data presented as Mean [Median] (SD) for continuous and *n* (%) for count data.

Characteristic	Bone level			Tissue level		
	All (*n* = 166)	3.3 mm (*n* = 83)	4.1 mm (*n* = 83)	All (*n* = 166)	3.3 mm (*n* = 83)	4.1 mm (*n* = 83)
Crestal bone level (mm)	0.75 [0.19] (1.47)	0.70 [0.18] (1.44)	0.81 [0.20] (1.50)	0.33 [0.09] (1.31)	−0.03 [−0.41] (1.27)	0.69 [0.54] (1.25)
Prosthetic platform height	1.52 [1.23] (0.91)	1.53 [1.20] (0.94)	1.52 [1.24] (0.89)	1.94 [1.80] (0.35)	1.80 [1.80] (0)	2.09 [1.80] (0.46)
Anatomical CIR	1.19 [1.15] (0.23)	1.10 [1.07] (0.18)	1.28 [1.26] (0.25)	0.89 [0.89] (0.22)	0.91 [0.89] (0.21)	0.87 [0.90] (0.22)
Clinical CIR	1.39 [1.27] (0.45)	1.30 [1.18] (0.42)	1.49 [1.38] (0.46)	1.35 [1.26] (0.52)	1.27 [1.21] (0.53)	1.42 [1.33] (0.50)
Emergence angle (°)						
Level 1	26 [26] (7)	24 [25] (8)	27 [27] (6)	7 [6] (7)	0 [0] (0)	15 [15] (1)
Level 2	16 [15] (8)	15 [15] (9)	16 [15] (8)	25 [23] (12)	27 [25] (12)	23 [20] (12)
Emergence profile level 1						
Concave	210 (63.3%)	100 (60.2%)	110 (66.3%)	166 (50.0%)	0 (0.0%)	166 (100.0%)
Convex	12 (3.6%)	10 (6.0%)	2 (1.2%)	0 (0.0%)	0 (0.0%)	0 (0.0%)
Straight	110 (33.1%)	56 (33.7%)	54 (32.5%)	166 (50.0%)	166 (100.0%)	0 (0.0%)
Emergence profile level 2						
Concave	82 (25.0%)	33 (20.2%)	49 (29.7%)	74 (22.6%)	45 (27.4%)	29 (17.8%)
Convex	125 (38.1%)	63 (38.7%)	62 (37.6%)	138 (42.2%)	61 (37.2%)	77 (47.2%)
Straight	117 (35.7%)	67 (41.1%)	50 (30.3%)	112 (34.3%)	58 (35.4%)	54 (33.1%)
Mixed	4 (1.2%)	0 (0.0%)	4 (2.4%)	3 (0.9%)	0 (0.0%)	3 (1.8%)
Peri‐implant diagnosis						
Health	35 (21.1%)	20 (24.1%)	15 (18.1%)	34 (20.5%)	20 (24.1%)	14 (16.9)
In conjunction to PIS	3 (1.8%)	1 (1.2%)	2 (2.4%)	5 (3.0%)	2 (2.4%)	3 (3.6%)
Peri‐implant Mucositis	112 (67.5%)	55 (66.3%)	57 (68.7%)	117 (70.5%)	59 (71.1%)	58 (69.9%)
In conjunction to PIS	17 (10.2%)	10 (12%)	7 (8.4%)	13 (7.8%)	7 (8.4%)	6 (7.2%)
Peri‐implantitis	19 (11.4%)	8 (9.6%)	11 (13.3%)	15 (9.0%)	4 (4.8%)	11 (13.3%)
In conjunction to PIS	5 (3.0%)	3 (3.6%)	2 (2.4%)	0 (0.0%)	0 (0.0%)	0 (0.0%)

Abbreviation: PIS, Peri‐implant sheathing.

**TABLE 2:** Inter‐rater agreement for crestal bone level and emergence angles including 95% confidence intervals.

Parameter	ICC
CBL	0.89 (0.87, 0.90)
Emergence angle level 1	0.98 (0.98, 0.98)
Emergence angle level 2	0.92 (0.91, 0.93)

Abbreviation: ICC, Intraclass correlation coefficients.

### 3.2 Associations Between Implant Design, Diameter, and Transmucosal Emergence Geometry

In the multivariate analysis, TL implants exhibited significantly narrower emergence angles than BL implants at L1 (−15.5°), whereas at L2 the angle was significantly wider (+6.6°; all *p* < 0.0001). A similar pattern was observed for implant diameter, with larger diameters associated with wider angles at L1 (+5.3°), but significantly narrower angles at L2 (−2.0°; all *p* ≤ 0.007). Furthermore, platform height was significantly associated with emergence angles at L1 (*p* < 0.0001), but not at L2 (*p* = 0.74). At L1, each 1 mm increase in platform height corresponded to a mean angular reduction of −2.6° (Table S2).

### 3.3 Associations of Peri‐Implant Disease Prevalence With Implant Characteristics and Transmucosal Emergence Geometry

**FIGURE 3:**
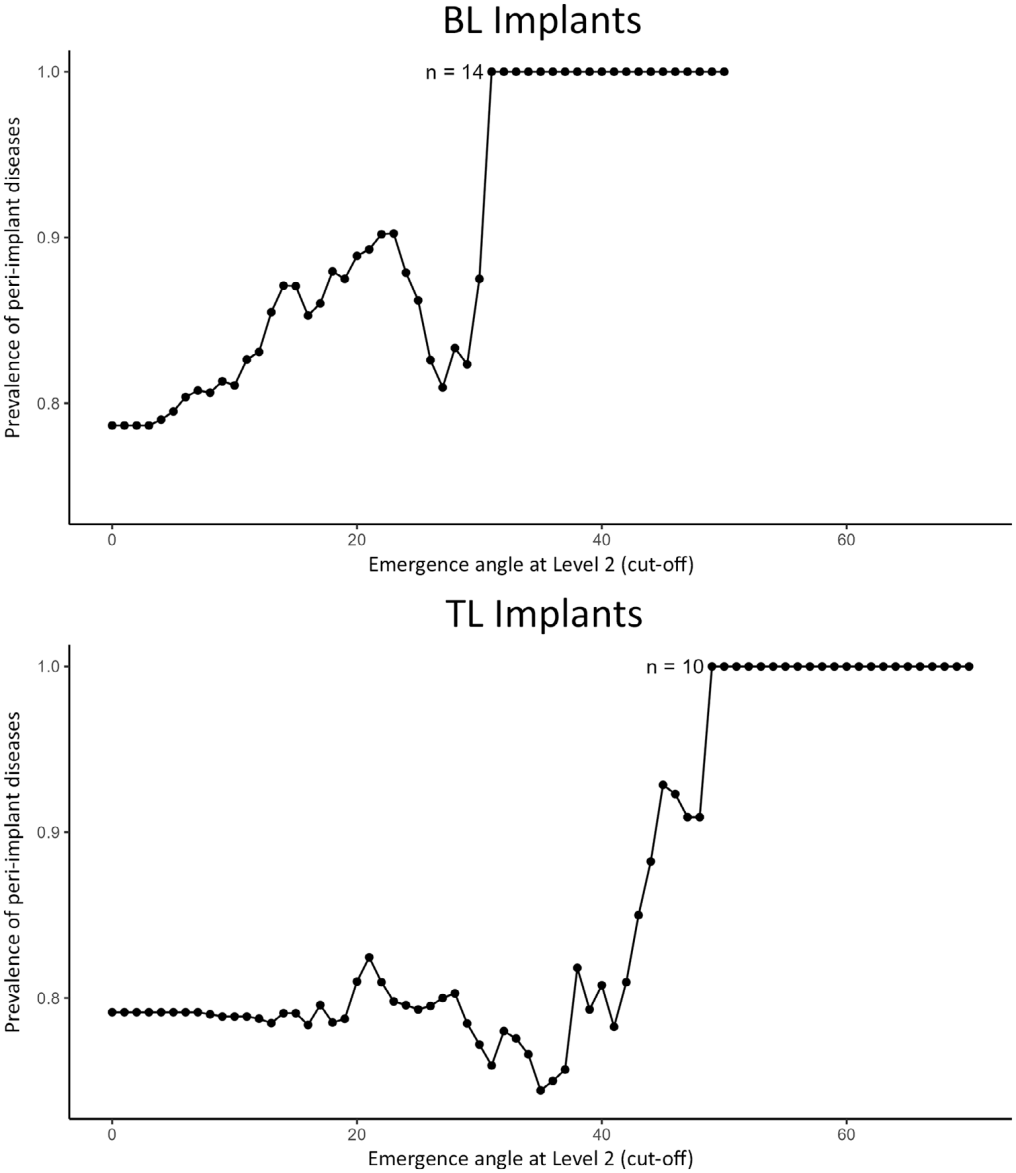
Association between peri‐implant disease prevalence and emergence angle at Level 2 (L2). The greater of the mesial and distal angles was used to assess how peri‐implant diseases change as the emergence angle is gradually increased and applied as a cut‐off point, that is, only implants with angles equal to or greater than the corresponding cut‐off were included. BL, bone‐level implant; TL, tissue‐level implant.

In the multivariate analysis, the implant diameter (OR 3.82, *p* < 0.0001) was significantly associated with peri‐implantitis, and the emergence angle at L2 (OR 1.03, *p* = 0.01) was significantly associated with peri‐implant mucositis (Table 3). When analyzing the proportion of implants affected by peri‐implant diseases relative to the emergence angle at L2, it was observed that BL and TL implants with emergence angles > 30° BL and > 48°, respectively, were affected by peri‐implant diseases (Figure 3). Other parameters did not reveal statistical significance; see univariate analyses (Table S3).

**TABLE 3:** Multivariate analysis for peri‐implant diseases (peri‐implant mucositis, peri‐implantitis) of selected parameters.

	OR	*p*
Peri‐implant mucositis		
Emergence angle level 2	1.03 (1.01; 1.05)	0.01*
Platform height	0.96 (0.72; 1.28)	0.78
Anatomical CIR	1.17 (0.54; 2.53)	0.69
Peri‐implantitis		
Diameter		< 0.0001***
3.3 mm	Baseline	
4.1 mm	3.82 (2.01; 7.23)	
Platform height	0.87 (0.57; 1.34)	0.54
Anatomical CIR	0.44 (0.14; 1.39)	0.16

*p < 0.05. ***p < 0.001.

### 3.4 Associations of CBL With Implant Characteristics and Transmucosal Emergence Geometry

Multivariate analyses revealed that mean CBL was significantly lower in TL compared to BL implants with a modeled effect of −0.24 mm (*p* = 0.036), and a significant difference between females and males (−0.27 mm, *p* = 0.029). CBL also appeared to be influenced by each additional 1 mm increase in platform height (−0.175 mm/mm, *p* = 0.01). Implant diameter (*p* = 0.24) and anatomical crown‐implant ratio were not significantly associated with CBL (*p* = 0.351, Table 4).

**TABLE 4:** Multivariate analysis for crestal bone levels of selected parameters for an average patient aged 60 with anatomical crown‐implant‐ratio (aCIR) = 1 and platform height = 1.8 mm.

	Effect	*p*
Intercept	0.50 (0.17; 0.83)	
Sex		0.02*
Male	Baseline	
Female	−0.27 (−0.50; −0.04)	
Implant design		0.036*
Bone level	Baseline	
Tissue level	−0.24 (−0.46; −0.02)	
Diameter		0.24
3.3 mm	Baseline	
4.1 mm	+0.12 (−0.08; 0.33)	
aCIR	−0.12 (−0.49; 0.24)	0.51
Emergence profile level 1		0.16
Concave	Baseline	
Convex	+0.32 (−0.37; 1.01)	
Straight	−0.16 (−0.36; 0.05)	
Platform height	−0.17 (−0.31; −0.04)	0.01**

*p < 0.05. **p < 0.01.

**FIGURE 4:**
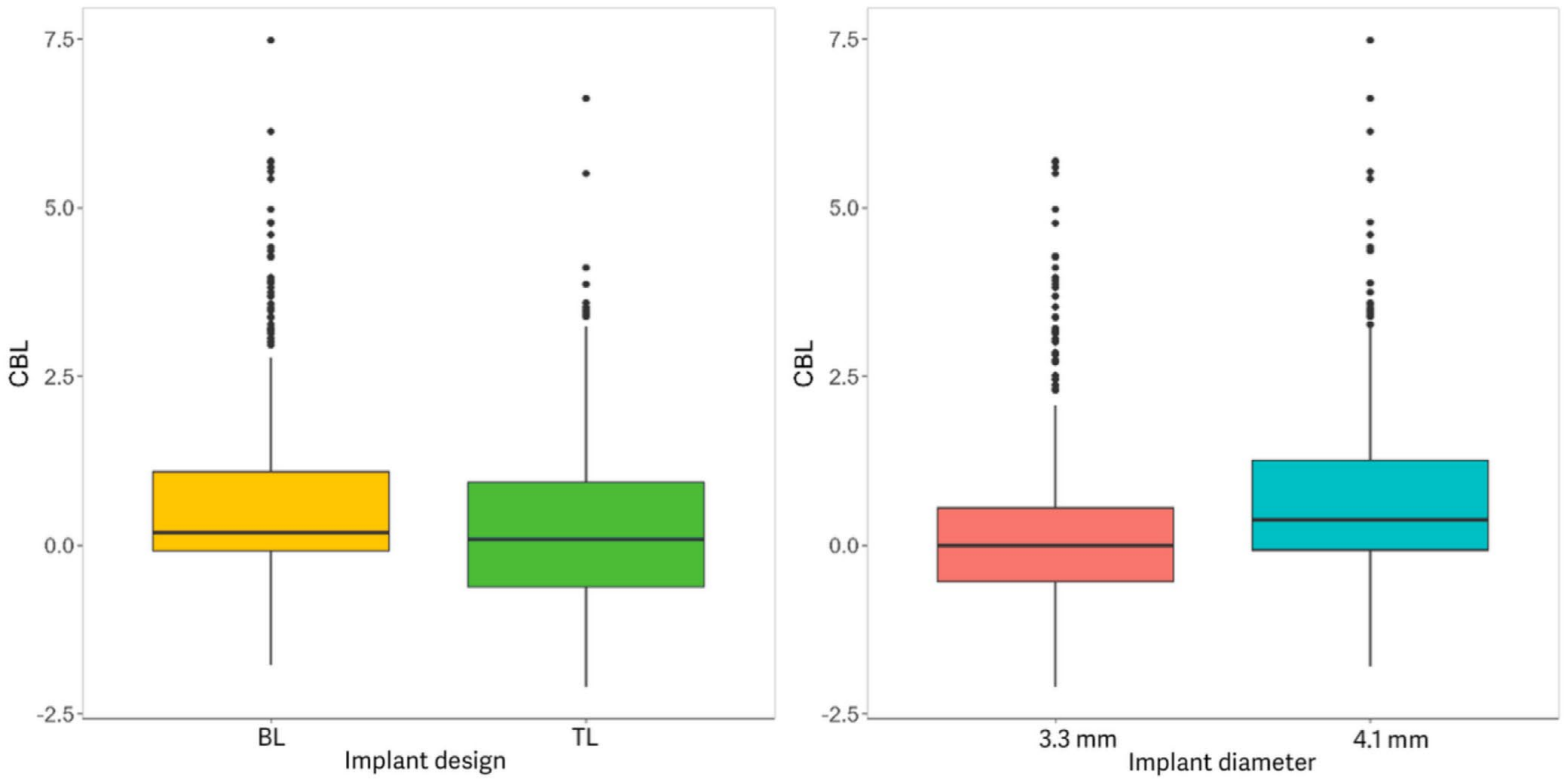
Boxplots depicting the distribution of CBL stratified by implant design and diameter. CBL, Crestal bone level; BL, bone‐level implant; TL, tissue‐level implant.

Furthermore, neither implant diameter (*p* = 0.24), nor profile type (*p* = 0.16), nor emergence angle at any level (*p* = 0.47, replacing profile type in Table 4) showed a significant association with CBL, despite significances in the univariate analyses, showing that their effects disappear when controlling for other factors. In the univariate analysis, wider emergence angles at L1 were significantly associated with increased CBL (*p* < 0.0001), whereas no effect was observed at L2 (*p* = 0.84). Mean CBL varied significantly according to the profile type at L1 (*p* < 0.0001), with convex profiles showing an effect of +0.323 mm compared to concave profiles (Supplementary Table 4). Finally, univariate analysis indicated that SDI was associated with a 0.22 mm increase in CBL compared to NDI (*p* = 0.002, Figure 4).

### 3.5 Associations With Other Radiographic Findings

Bone graft substitute remnants were detected in 19.9% of TL and 26.5% of BL implant sites, as well as in 19.9% of NDI and 26.5% of SDI sites. Cement remnants were identified in only one case (BL SDI). A misfit of the ISP was observed in 12.7% of TL and 9.6% of BL, as well as in 10.8% of NDI and 12.0% of SDI; however, no significant associations with peri‐implant health status were identified. Peri‐implant sheathing and corticalization were observed in 10.8% and 15.1% of TL vs. 15.1% and 21.1% of BL implants, and in 13.9% and 18.1% of NDI vs. 12.0% and 18.1% of SDI, respectively. However, no significant association between implant design and diameter was found (*p* ≥ 0.29). No signs of retrograde peri‐implantitis or implant fracture were observed. Details on the subgroup prevalence of radiographic findings are displayed in Table 5.

**TABLE 5:** Prevalences of radiographic findings depending on implant design and diameter.

	BL (*n* = 166)				TL (*n* = 166)			
	3.3 (*n* = 83)		4.1 (*n* = 83)		3.3 (*n* = 83)		4.1 (*n* = 83)	
	*n*	%	*n*	%	*n*	%	*n*	%
Augmentation particles in the peri‐implant soft tissues	18	21.7	26	31.3	15	18.1	18	21.7
Cement remnants	0	0.0	1	1.2	0	0	0	0
Misfit of ISP	9	10.8	7	8.4	9	10.8	13	15.7
Peri‐implant sheating	14	16.9	11	13.3	9	10.8	9	10.8
Peri‐implant corticalization	17	20.5	18	21.7	13	15.7	12	14.5
Retrograde periimplantitis	0	0	0	0	0	0	0	0
Implant fracture	0	0	0	0	0	0	0	0

Abbreviations: BL, Bone‐level implant; ISP, implant‐supported prosthesis; TL, Tissue‐level implant.

## 4 Discussion

This cross‐sectional study evaluated the association of transmucosal emergence geometry on the prevalence of peri‐implant diseases and CBL in single‐tooth, non‐molar BL and TL implants. Multivariate analyses demonstrated that wider emergence angles at L2 were significantly associated with peri‐implant diseases, whereas implant diameter was associated with peri‐implantitis prevalence. Furthermore, higher CBL was observed in BL implants, male patients, and ISPs with shorter platform heights.

In the present study, the prevalence of peri‐implant mucositis and peri‐implantitis was 70.5% and 9.0% for TL implants and 67.5% and 11.4% for BL implants, respectively. These values align with the 62.6% peri‐implant mucositis and 7.5% peri‐implantitis reported in a cross‐sectional study on subcrestally placed implants with a median time in function of 9.4 years (Obreja et al. 2021). In contrast, a recent meta‐analysis reported lower peri‐implant mucositis rates (59.2%) and higher peri‐implantitis rates (18.0%) (dos Reis et al. 2025). The discrepancy in peri‐implant mucositis prevalence may be partly explained by methodological differences during clinical examination, such as variations in probing force, as well as differences in the underlying patient populations. Conversely, the lower prevalence of peri‐implantitis observed in the present cohort may be attributable to comprehensive treatment planning and precise implant placement in prosthetically ideal positions, as typically achieved in a university‐based clinical setting. Additionally, details on peri‐implant conditions in this patient cohort have been previously reported (Couso‐Queiruga, Fonseca, et al. 2025). In the present study, peri‐implant disease prevalence varied as a function of transmucosal emergence geometry, consistent with previous reports (Katafuchi et al. 2018; Lin et al. 2025; Yi et al. 2020). However, emergence angles in these studies were assessed only at the implant–abutment interface, the position of which relative to the crestal bone differs between bone‐level and tissue‐level implant designs and may affect the observed associations. Therefore, the present investigation analyzed emergence angles at different levels considering implant and prosthetic characteristics. Notably, only wider emergence angles at L2 but not at L1 were associated with a greater prevalence of peri‐implant mucositis, highlighting the necessity for adequate ISP design in the transmucosal zone. Interestingly, visual inspection of the data distribution indicated that all BL implants with emergence angles > 30° and all TL implants with angles > 48° were associated with peri‐implant diseases. These critical emergence angle values for BL implants are consistent with those reported by Katafuchi et al. (2018). To the best of our knowledge, this is the first study to identify a threshold in TL implants beyond which peri‐implant diseases are invariably observed. Although these thresholds should be interpreted with caution as no ROC analysis was performed, they may be considered during the treatment planning process to guide the selection of implant fixture specifications, surgical execution (i.e., apico‐coronal implant position), and ISP design features, and to help identify existing ISPs at risk, thereby supporting the choice of appropriate long‐term implant maintenance strategies. The higher threshold observed in TL implants may be explained by the taller mean platform height, with a minimum platform height of 1 mm in BL compared to 1.8 mm in TL. It has been suggested that a greater vertical distance may reduce the extent of the inflammatory infiltrate by positioning the microgap further away from the crestal bone, enhancing the sealing of the supracrestal soft tissues and decreasing stress caused by a dysbiotic microbial biofilm and functional stress, even in the presence of marginal misfit at the implant‐superstructure interface (Couso‐Queiruga, Ramseier, et al. 2025; Spinato et al. 2018). Additionally, larger implant diameters were independently associated with a higher prevalence of peri‐implantitis, which has also been reported by other authors (Apaza‐Bedoya et al. 2024; González‐Valls et al. 2021; Pesce et al. 2023). A possible explanation could be the reduced peri‐implant phenotype dimensions around larger diameter implants, with thinner peri‐implant mucosa and bone potentially reducing peri‐implant tissue resilience to biological or functional challenges (Bressan et al. 2023; Imber et al. 2025; Monje et al. 2019). Interestingly, implant design was not associated with the overall peri‐implant disease prevalence, supporting the clinical applicability of both BL and TL implants.

Absence of radiographic bone loss remains a primary indicator of implant health, as its presence and progression may be associated with biological and functional complications. In this study, mean CBL was 0.54 ± 1.34 mm, with a trend toward lower CBL around TL implants. This finding is consistent with cross‐sectional and meta‐analytic evidence reporting greater bone stability around TL compared to BL implants (Elkattan et al. 2025; Katafuchi et al. 2018; Moser et al. 2026; Rokn et al. 2017). Therefore, while both implant designs are viable options, TL implants may offer biological advantages, especially in posterior sites where esthetics are not a priority. However, this contrasts with the findings of a systematic review that included studies with 1–5 years of follow‐up, which reported no significant differences in CBL between implant designs (Cosola et al. 2020). Regarding implant diameter, the univariate association between wider implant diameter and increased CBL was attenuated in the multivariate model (0.123 mm greater for SDIs), suggesting the influence of confounding factors related to the implant, the prosthesis, and/or the patient. This finding aligns with the results of a randomized controlled trial that reported higher CBL around BL SDIs compared to BL NDIs in single‐tooth sites within one year after implant loading (Ghazal et al. 2019). Our observations therefore support the clinical validity of NDI for appropriately selected indications, without an increased risk of CBL. Interestingly, transmucosal emergence geometry features were not independently associated with CBL in multivariate analyses but showed significance in univariate models, meaning that their effect is reduced or confounded when controlling for other possible influential covariates in the data set. This finding contrasts with studies identifying emergence angles > 30° as risk indicators for crestal bone loss around BL but not for TL implants (Yi et al. 2020). However, it is in agreement with the results of a meta‐analysis that did not find a relationship between transmucosal emergence geometry and crestal bone changes (Atieh et al. 2023).

Another relevant factor within the transmucosal zone is the prosthetic platform height. In our study, higher platform height was significantly associated with reduced CBL, with each additional millimeter resulting in a 0.175 mm reduction in CBL. This is consistent with previous reports and supports the existing evidence on the beneficial effect of transmucosal height beyond a certain threshold, which may contribute to a more robust biological seal, thereby limiting peri‐implant disease onset and progressive crestal bone loss (Apaza‐Bedoya et al. 2024; Del Amo et al. 2024; Lee, Kim, Kweon, and Kim 2018; Spinato et al. 2018). Another study reported on a synergistic effect: emergence angle impacts bone stability only when platform height is < 2 mm in BL implants, reinforcing the need for adequate supracrestal tissue height to accommodate prosthetic contours while avoiding crestal bone encroachment (Misch et al. 2025). Therefore, the design of the ISP in proximity to the crestal bone is critical and should be meticulously considered. Moreover, maintaining sufficient supracrestal soft tissue height is equally critical to ensure long‐term stability and biological sealing (Puisys et al. 2024). When restoring BL implants, taller abutments and narrower emergence angles may offer biological advantages, an aspect inherently accounted for in TL implants due to their integrated transmucosal design. Interestingly, sex was another significant factor affecting CBL, with females exhibiting lower CBL than males, an observation rarely reported in the literature. Potential contributing factors to this observation may include lower occlusal forces, a reduced prevalence of periodontitis, and lower smoking rates among females. Moreover, male sex has been associated with higher implant failure rates, possibly due to hormonal differences, variations in bone density, and behavioral factors such as oral hygiene and compliance (Tobias et al. 2025).

Beyond changes in CBL resulting from initial physiologic remodeling after insertion of the final prosthesis, crestal bone loss may also display as a linear peri‐implant sheathing (i.e., a linear peri‐implant radiolucency) accompanied by corticalization of the surrounding bone in the absence of clinical signs or symptoms. This radiographic pattern may reflect a loss of osseointegration other than peri‐implantitis, a phenomenon that remains poorly understood and potentially influenced by implant‐related factors such as implant design, length and diameter due to differences in stress distribution to the crestal bone (Biel et al. 2022; Raabe et al. 2021). In the present study, peri‐implant sheathing and corticalization were observed in 10.8% to 15.1% of cases, respectively, irrespective of implant design or diameter. It is important to acknowledge that evidence on peri‐implant sheathing remains scarce, and at present, these radiographic findings are not classified as distinct disease entities within the EFP/World Workshop framework and lack a validated diagnostic and prognostic framework (Berglundh et al. 2018; Herrera et al. 2023). Additionally, no cases of retrograde peri‐implantitis were identified. In contrast to crestal inflammatory processes, retrograde peri‐implantitis presents as a distinct apical lesion, typically associated with residual endodontic pathology or surgical trauma, while the crestal bone remains stable (Di Murro et al. 2021). Furthermore, no implant fractures were observed in this investigation. This aligns with findings from a multicenter long‐term study reporting a very low fracture rate of 0.4%, which may be associated with bone loss and implant manufacturing defects (Lee, Kim, Jeong, et al. 2018).

The present study is not without limitations. First, the cross‐sectional design does not allow for establishing causal relationships and may be subject to selection bias due to retrospective patient recruitment. A longitudinal follow‐up would be needed to confirm the progression and risk factors over time. As patients were not randomly assigned to receive either BL or TL implants, nor to implant diameters, there is a risk of confounding the results by indication or clinical judgment. Additionally, the academic single‐center and exclusion of molar sites limit generalizability to other clinical settings, such as private practices with differing implant location, treatment protocols, implant systems, levels of operator experience, and patient populations. Second, peri‐implant diseases were analyzed as a binary outcome, and continuous CBL measurements were not used for disease severity stratification, which may underestimate the clinical heterogeneity of these conditions. Third, the biological response at the implant–abutment interface may be influenced not only by implant design but also by the properties of the restorative materials (e.g., surface roughness), which may act as confounding variables. Fourth, the inclusion of only surviving implants at follow‐up may introduce survivor bias. Consequently, the reported prevalence of complications may be underestimated, and the findings should be interpreted as representative of surviving implants rather than the entire cohort originally treated. Finally, future studies should use a prospective longitudinal design to assess the onset, progression, and temporal dynamics of peri‐implant diseases and crestal bone loss in relation to implant location, design, diameter, and prosthetic‐related characteristics over time. A randomized allocation of different implant designs and diameters would help isolate the possible causal effects of macro‐design and implant‐abutment connection on implant‐related outcomes, therefore reducing bias.

## 5 Conclusions

Within the limitations of this study, it can be concluded that:

– Crown emergence angles > 30° in BL implants and > 48° in TL implants are predictors of peri‐implant diseases.
– Wider implant diameters were associated with an increased peri‐implantitis prevalence.
– TL implants and greater platform height were associated with reduced CBL.
– Radiographic peri‐implant sheathing and corticalization were common findings but appeared independent of implant design.

## Author Contributions

**Clemens Raabe:** conceptualization, methodology, software, data curation, supervision, formal analysis, resources, project administration, writing – review and editing, visualization, investigation, funding acquisition, writing – original draft, validation. **Manrique Fonseca:** writing – review and editing, investigation. **Vivianne Chappuis:** writing – review and editing, resources. **Gustavo Avila‐Ortiz:** writing – review and editing. **Diogo Moreira Rodrigues:** writing – review and editing. **Pablo Galindo‐Moreno:** writing – review and editing. **Emilio Couso‐Queiruga:** conceptualization, methodology, data curation, software, formal analysis, validation, visualization, writing – review and editing, investigation, funding acquisition, writing – original draft.

## Funding

This study was supported by a research grant from the International Team for Implantology (ITI Grant No. 1905‐2024).

## Disclosure

The authors used ChatGPT (OpenAI) for language editing during manuscript preparation, and all generated text was carefully reviewed and revised by the authors, who assume full responsibility for the final content.

## Ethics Statement

This study has been independently reviewed and approved by the committee of the state of Bern, Switzerland (ID 2023‐01651).

## Conflicts of Interest

The authors declare no conflicts of interest.

## Supporting information


**Table S1:** Summary of sex, age, implant location, surgical procedure and ISP retention by implant design and diameters.
**Table S2:** Multivariate analysis regarding emergence angle at Levels 1 and 2 for an average patient aged 60 years with anatomical CIR = 1 and platform height = 1.8.
**Table S3:** Univariate tests for peri‐implant diseases (peri‐implant mucositis, peri‐implantitis) for selected parameters.
**Table S4:** Univariate tests for crestal bone level (CBL).

**Data S1:** STROBE Statement—checklist of items that should be included in reports of observational studies.

## Data Availability

All data related to this study are available upon reasonable request to the corresponding author.
