# Supplementary material for: Association of Transmucosal Emergence Geometry and Peri‐Implant Diseases Prevalence Around Bone‐ and Tissue‐Level Implants: A Cross‐Sectional Study
Source: Clin Oral Implants Res. 2026 Apr 12;37(7):883–95. doi: 10.1111/clr.70131 (PMC13340502; doi:10.1111/clr.70131)
Supplement: Supplementary file 1 — Table S1: Summary of sex, age, implant location, surgical procedure and ISP retention by implant design and diameters. Table S2: Multivariate analysis regarding emergence angle at Levels 1 and 2 for an average patient aged 60 years with anatomical CIR = 1 and platform height = 1.8. Table S3: Univariate tests for peri‐implant diseases (peri‐implant mucositis, peri‐implantitis) for selected parameters. Table S4: Univariate tests for crestal bone level (CBL). [file CLR-37-883-s001.docx]

**Supplementary Tables**

|  | **Bone Level** | | | **Tissue Level** | | | |
| --- | --- | --- | --- | --- | --- | --- | --- |
| **Characteristic** | **All (n=166)** | **3.3 mm (n=83)** | **4.1 mm (n=83)** | **All (n=166)** | **3.3 mm (n=83)** | **4.1 mm (n=83)** |  |
| **Age** | **55 [58] (16.2)** | **52.5 [53] (17.7)** | **57.5 [61] (14.1)** | **66 [70] (15.1)** | **64.5 [69] (15.9)** | **67.4 [71] (14.1)** |  |
| **Sex** |  |  |  |  |  |  |  |
| **Male** | **79 (47.6%)** | **46 (55.4%)** | **33 (39.8%)** | **88 (53.0%)** | **49 (59.0%)** | **39 (47.0%)** |  |
| **Female** | **87 (52.4%)** | **37 (44.6%)** | **50 (60.2%)** | **78 (47.0%)** | **34 (41.0%)** | **44 (53.0%)** |  |
| **Jaw** |  |  |  |  |  |  |  |
| **Maxilla** | **147 (88.6%)** | **66 (79.5%)** | **81 (97.6%)** | **108 (65.1%)** | **53 (63.9%)** | **55 (66.3%)** |  |
| **Mandible** | **19 (11.4%)** | **17 (20.5%)** | **2 (2.4%)** | **58 (34.1%)** | **30 (36.1%)** | **28 (33.7%)** |  |
| **Implant Location** |  |  |  |  |  |  |  |
| **Central Incisor** | **63 (38.0%)** | **9 (10.8%)** | **54 (65.1%)** | **13 (7.8%)** | **7 (8.4%)** | **6 (7.2%)** |  |
| **Lateral Incisor** | **58 (34.9%)** | **54 (65.1%)** | **4 (4.8%)** | **16 (9.6%)** | **16 (10.8%)** | **0 (0.0%)** |  |
| **Canine** | **12 (7.2%)** | **4 (4.8%)** | **8 (9.6%)** | **6 (3.6%)** | **3 (3.6%)** | **3 (3.6%)** |  |
| **First Premolar** | **19 (11.4%)** | **7 (8.4%)** | **12 (14.5%)** | **56 (33.7%)** | **28 (33.7%)** | **28 (33.7%)** |  |
| **Second Premolar** | **14 (8.4%)** | **9 (10.8%)** | **5 (6.0%)** | **75 (45.2%)** | **29 (34.9%)** | **46 (55.4%)** |  |
| **Surgical Procedure** |  |  |  |  |  |  |  |
| **Standard Implant Placement** | **20 (12.0%)** | **13 (15.7%)** | **7 (8.4%)** | **50 (30.1%)** | **19 (22.9%)** | **31 (37.3%)** |  |
| **Simultaneous HBA** | **141 (84.9%)** | **68 (81.9%)** | **73 (88.0%)** | **92 (55.4%)** | **54 (65.1%)** | **38 (45.8%)** |  |
| **Staged HBA** | **4 (2.4%)** | **2 (2.4%)** | **2 (2.4%)** | **4 (2.4%)** | **3 (3.6%)** | **1 (1.2%)** |  |
| **SFE** | **4 (2.4%)** | **1 (1.2%)** | **3 (3.6%)** | **26 (15.7%)** | **10 (12.0%)** | **16 (19.3%)** |  |
| **ISP** |  |  |  |  |  |  |  |
| **Cemented** | **12 (7.2%)** | **6 (7.2%)** | **6 (7.2%)** | **36 (21.7%)** | **9 (10.8%)** | **27 (32.5%)** |  |
| **Screw-retained** | **154 (92.8%)** | **77 (92.8%)** | **77 (92.8%)** | **127 (76.5%)** | **71 (85.5%)** | **56 (67.5%)** |  |

Supplementary Table 1 Summary of sex, age, implant location, surgical procedure and ISP retention by implant design and diameters. Data presented as Mean [Median] (SD) for continuous and n (%) for count data. ISP: Implant-supported prosthesis. HBA: horizontal bone augmentation. SFE: sinus floor elevation.

| **Level** | **Emergence Angle Level 1** | | **Emergence Angle Level 2** | |
| --- | --- | --- | --- | --- |
|  | **Effect** | **p-value** | **Effect** | **p-value** |
| **Intercept** | **25.4 (24.8; 25.9)** |  | **16.9 (13.8; 20.0)** |  |
| **Sex** |  | **1.00** |  | **0.16** |
| **Male** | **Baseline** |  | **Baseline** |  |
| **Female** | **0.0 (-0.6; 0.6)** |  | **-1.1 (-2.7; 0.4)** |  |
| **Implant Design** |  | **<0.0001***** |  | **<0.0001***** |
| **Bone Level** | **Baseline** |  | **Baseline** |  |
| **Tissue Level** | **-15.5 (-16.0; -15.0)** |  | **+6.6 (4.7; 8.5)** |  |
| **Diameter** |  | **<0.0001***** |  | **0.007**** |
| **3.3mm** | **Baseline** |  | **Baseline** |  |
| **4.1mm** | **+5.3 (4.9; 5.8)** |  | **-2.0 (-3.5; -0.6)** |  |
| **Anatomical CIR - 1** | **-0.3 (-1.1; 0.4)** | **0.41** | **-3.1 (-6.5; 0.3)** | **0.07**** |
| **Platform height – 1.8** | **-2.6 (-2.9; -2.3)** | **<0.0001***** | **-0.2 (-1.2; 0.9)** | **0.74** |

Supplementary Table 2 Multivariate analysis regarding emergence angle at Levels 1 and 2 for an average patient aged 60 years with anatomical CIR = 1 and platform height = 1.8. * p <0.05. ** p<0.01. *** p<0.001.

|  | **OR** | **p-value** |
| --- | --- | --- |
| **Peri-implant Mucositis** |  |  |
| Implant Design |  | 0.89 |
| Bone Level | Baseline |  |
| Tissue Level | 1.04 (0.58; 1.86) |  |
| Diameter |  | 0.23 |
| 3.3mm | Baseline |  |
| 4.1mm | 1.44 (0.79; 2.61) |  |
| Emergence Angle |  |  |
| Level 1 |  | 0.69 |
| Level 2 |  | 0.03* |
| Emergence profile |  |  |
| Level 1 |  | 0.14 |
| Level 2 |  | 0.46 |
| **Peri-implantits** |  |  |
| Implant Design |  | 0.71 |
| Bone Level |  |  |
| Tissue Level | Baseline |  |
| Diameter | 0.84 (0.34; 2.07) | 0.01* |
| 3.3mm |  |  |
| 4.1mm | Baseline |  |
| Emergence Angle | 2.20 (1.19; 4.07) |  |
| Level 1 |  | 0.18 |
| Level 2 |  | 0.50 |
| Emergence Profile |  | 0.11 |
| Level 1 |  | 0.11 |
| Level 2 |  | 0.39 |

Supplementary Table 3 Univariate tests for peri-implant diseases (peri-implant mucositis, peri-implantitis) for selected parameters. * p <0.05. ** p<0.01. *** p<0.001.

|  | **Effect** | **p-value** |
| --- | --- | --- |
| **Implant Design** |  | **<0.0001***** |
| **Bone Level** | **Baseline** |  |
| **Tissue Level** | **-0.36 (-0.53; -0.19)** |  |
| **Diameter** |  | **0.002**** |
| **3.3mm** | **Baseline** |  |
| **4.1mm** | **+0.22 (0.08; 0.36)** |  |
| **Emergence Angle** |  |  |
| **Level 1** |  | **<0.0001***** |
| **Level 2** |  | **0.84** |
| **Emergence Profile** |  |  |
| **Level 1** |  | **<0.0001** |
| **Level 2** |  | **0.19** |

Supplementary Table 4 Univariate tests for crestal bone level (CBL). * p <0.05. ** p<0.01. *** p<0.001.
